# Editorial: Network Spread Models of Neurodegenerative Diseases

**DOI:** 10.3389/fneur.2018.01159

**Published:** 2019-01-08

**Authors:** Ashish Raj, Yasser Iturria-Medina

**Affiliations:** ^1^Department of Radiology, Weill Cornell Medicine, New York, NY, United States; ^2^Department of Radiology, University of California at San Francisco, San Francisco, CA, United States; ^3^Montreal Neurological Institute, McGill University, Montreal, QC, Canada

**Keywords:** connectome, neurodegeneration, graph theory, Alzheimer's disease, protein aggregation, trans-neuronal transmission

The emerging field of network neuroscience visualizes the brain as a graph consisting of nodes representing regions and edges as connections between them. This complex network supports efficient communication along neural projections, but also, unfortunately, the transmission and progression of neurodegenerative disorders like Alzheimer's disease (AD). If we could know the brain's network organization, could we then predict how degenerative processes might develop on this network? The answer is, increasingly, yes. This Research Topic collects a series of papers on various network models of dementia spread, focusing on disease-specific mathematical modeling rather than general graph theory.

## Modeling Neurodegenerative Dynamics on Brain Networks

Disturbances in global and local network organization are well documented in neurodegenerative diseases including AD (1), frontotemporal dementia (FTD) (2) and amyotrophic lateral sclerosis (ALS) (3). However, far more interesting than static networks is the potential to understand their dynamics—ongoing brain changes throughout disease (4). Broadly, this can happen in two ways: *First*, aberrant connectivity and *network degeneration* (5–7) via demyelination and axonal injury, secondary Wallerian degeneration, loss of signaling, axonal, and dendrite retraction. *Second*, disease factors can directly propagate along (possibly unchanging) neural connections, underpinned by “prion-like” protein aggregation followed by their trans-synaptic transmission (8–14). Thus, instead of being primarily impaired in degeneration, the network serves mainly as a conduit for disease transmission. Which type of dynamics predominates in neurodegeneration is a matter of lively debate.

In this Issue, Carbonell et al. provide a condensed historical review summarizing mathematical modeling of complex misfolded proteins mechanisms, both at the local/regional level and at the whole brain network level. They describe many recent network spread models, including models of cooperative spread (15), and communication cascades (16). In particular, an epidemic spreading model of network spread (17) was successfully validated on PET Amyloid-ß patterns in AD patients. An analytical Network Diffusion Model (NDM) mathematically derived the behavior of protein transmission as a graph heat equation under a connectivity-driven mechanism (18). This model is the basis of 2 papers in this Topic–Mezias and Raj and Pandya et al.

The accompanying paper by Mezias and Raj set out to apply the NDM, originally used on human data, to the problem of predicting the progression of Aβ pathology in transgenic mouse models. They show that while NDM is capable of predicting Aβ spread, it is not necessarily better than a model of spatial spread—one that does not involve network transmission. This is in contrast to the same authors' earlier publication showing a strong network mediation effect of tau spread (19), revealing a potentially important distinction between their respective mechanisms.

Pathological commonality and overlap is observed in various dementias: misfolded *tau*, A-*beta* and alpha-synuclein are present to varying degree in most degenerative diseases: AD, semantic dementia, FTD (20), ALS, dementia with Lewy bodies and posterior cortical atrophy (21). Pandya et al. explore this issue in a rare tauopathy called Progressive Supranuclear Palsy (PSP), which principally affects brainstem and striatal areas. Using the NDM Pandya et al. were able to recapitulate empirical PSP atrophy patterns. Although human imaging does not enable measurement of projection polarity, they presented a cross-species approach that transfers some directionality information from mouse to homologs regions in humans. Using this directional connectome, they found that anterograde and retrograde transmission give somewhat different spatiotemporal patterns of spread.

This raises important questions that can only be resolved by future advances in connectomics. Interestingly, the contributing paper by Neitzel et al. addresses this point directly in their perspective article. Using multimodal imaging data, including PET and functional MRI, they give a new perspective on how these techniques can be used to infer directionality of network connections in the human brain. As and when these techniques become increasingly refined and widely adopted, we predict that the issue of pathology spread on directional networks might assume critical ramifications.

The paper by Manuello et al. takes a meta-analysis approach for analyzing voxel-based morphometry data to understand the phenomenon of network spread. They report that in AD, gray matter alterations do not occur randomly across the brain but, on the contrary, follow identifiable patterns of distribution. This alteration pattern exhibits a network-like structure composed of co-altered areas that can be defined as co-atrophy network. The benefit of this type of data-driven approach is that it does not rely on specific hypotheses about network spread, unlike the papers by Mezias and Raj and Pandya et al.

A data-driven methodology was also adopted by Koval et al. which used the temporal alignment and combination of several short-term observation data to reconstruct the long-term atrophy history of AD. This model provided a description of both the spatiotemporal patterns of cortical atrophy at the group level and the variability of these patterns at the individual level in terms of propagation pathways, speed of propagation, and age at propagation onset.  Oxtoby et al. explored how the pathology propagates through the connectivity network via a data-driven event-based model. By analyzing the changes in the elderly brain's anatomical connectivity over the course of AD, the authors clarified both the location and the sequence of changes to white matter connections. The results supported that degeneration of anatomical connectivity in the human brain may be an early and valuable biomarker when studying neurodegenerative diseases. Two other data-driven studies,  Weber et al. and Huang et al., illustrate the methodological importance of considering focal axonal swelling and functional connectivity analysis for quantifiying memory deterioration rates in traumatic brain injury and evaluating clinical effects in infarction patients with dysphagia, respectively.

## Summary and Outlook

The present contributions encompass several cutting-edge techniques for investigating the phenomenon of networked spread in neurodegeneration, both model-based and data-driven. Together, they provide a framework for understanding the way the disease moves around within the brain network, that might adequately explain archetypal patterns of regional specificity in various dementias. Moving away from current statistical or descriptive graph theory, these papers trace underlying network dynamical processes. The emerging frontier of network spread modeling has the potential for wide applicability in diagnostics and therapeutic interventions.

## Author Contributions

All authors listed have made a substantial, direct and intellectual contribution to the work, and approved it for publication.

### Conflict of Interest Statement

The authors declare that the research was conducted in the absence of any commercial or financial relationships that could be construed as a potential conflict of interest.
